# Modern Drug Laboratories

**Published:** 1887-12-03

**Authors:** 


### Modern Drug Laboratories.

By our Special Commissioner.

(*Continued from page* 130, *vol. iii.*)

It is natural that dwellers in foreign countries, where there is not a drug-shop in every street, travellers, and explorers, should value these compressed medicines. If you live in a country where snake-bite is one of the ills that flesh is heir to, it is very convenient to have your permanganate of potash in such a form that you can take it about with you and never know, from any trouble its transit causes you, that you have it, yet find it at hand and ready for use when you need it. For explorers, who are for many months out of reach of aid from civilisation, a medicine being supplied in a portable form often means the possibility of carrying a sufficient quantity, a thing perhaps involving life or death. It is wonderful how much material may be put into a a small medicine chest if the drugs be thus compressed and concentrated. Of this the "Congo" medicine chest, with which Stanley supplied himself, is a remarkable proof. As it is meant to be carried on a man's head or shoulders, it is necessary that it should be neither large nor heavy; but it is made of strong metal, clasped with brass, and provided with locks that would puzzle the average thief, and contains forty bottles—a pretty good store if each is filled with the concentrated form of a drug. In the lid are compartments for surgical instruments, lint, and bandages; and as the bottles are each wrapped in fibre lint, and packed in absorbent cotton, every particle of space is usefully filled.

The tabloids, however, which are likely to be most popular in this country are not as yet incorporated into the pharmacopœia. They are made of saccharine, one of the most remarkable products of coal-tar. To enumerate the many and elaborate processes by which it is evolved would take up too much room here to relate. It is sufficient to say that benzoyl sulphonic imide—or saccharine, as it is called in commerce—is a white powder with an intensely sweet taste, but is, nevertheless, not a sugar; that is, it is not nutritive, does not ferment, has none of the chemical actions of sugar, while its sweetening power is infinitely greater; therefore it is of great value in diseases where sugar is injurious. A doctor is placed in a difficult position when, for example, a patient suffering from diabetes complains that he "can't get on" without sugar in his tea or coffee, sweet puddings, &c. In such a dilemma saccharine comes to the aid. The patient's appetite can be satisfied without injury to his health. He can have all his food as sweet as he likes; indeed, if he has not accurately informed himself of the relative sweetening powers of cane-sugar and saccharine, he will probably make it much sweeter than he intends, for saccharine has three hundred times the power of sugar, and a tablet about half the size of a pea is equal to a pretty large lump of sugar.

Worthy of mention, too, is the preparation of strophanthus, a lately discovered remedy for heart disease, which is in many cases superseding digitalis. Strophanthus is a plant of the periwinkle order, and is a native of Central Africa. It was originally known as a poison, the "kombé" arrow poison of which Livingstone speaks, and Professor Fraser devoted fifteen years to experiments on the plant before he announced to the British Medical Association in 1885 his belief in its curative value. The tincture is obtained from the seeds, which are imbedded in the walls of pods about 12 inches long. At first it was difficult to obtain sufficient quantities of the plant, as much as £20 being given for one pound of the seeds; but now that its value is known, it is being extensively cultivated, and the growth of the once-dreaded strophanthus forms one of the industries of the mission station at Blantyre.

The packing-room of the factory is one of the most pleasant in the place. It is a long, light, well-ventilated room, very plainly furnished (with certain exceptions to be noted hereafter) with long deal tables and benches, about which are clustered a number of girls in groups of two or three, engaged in some part of the finishing touches which prelude the appearance of a bottle of medicine in the market. One fits the cork in a bottle of Malt Extract, cutting off any superfluity with a large knife before passing it to another, who washes off any possible stickiness with tepid water, and then passes it along to a third, who fits on a tin capsule by an ingenious arrangement of double circles of string. Then comes the labelling, each label being stamped with a special letter and number, in order to facilitate inquiry in case of any complaint regarding the quality or quantity of the contents of the bottle. Then it is wrapped round in a paper of instructions printed in the language of the country to which it is to go—when I passed through, a consignment was being prepared for South America, with the necessary advice in Spanish—and afterwards fastened up in a neat pasteboard case, in company with a small corkscrew, just strong enough to open the bottle; so that there is saved that bungling over the administration of the dose which often makes a patient loathe his medicine before he tries it. On the other side girls are filling bottles with a fine grey powder—pancreatine, the friend of weak digestions. The corks which stop these bottles form at one end a five-grain measure, so that any inaccuracy in the quantity of the dose is inexcusable. Others are weighing tabloids, and putting them into the boxes or tubes in which they are to be sent out. All are busy at work which is well within the compass of their powers; all seem happy.

I said there were some exceptions to the plainness of the furnishing. One of these is a piano, intended for the use of the employées, supported by a goodly pile of magazines for those whose tastes tend more to literature than to music. In a recess is seen a large array of teacups, etc., while a kettle boiling on a gas-stove at one end of one room suggests a prospect of tea. Consideration and courtesy towards those who work for them are among the rules of this firm, the result being that the masters are regarded with a friendly feeling and loyalty very rare in these days, when the wrongs of the working man, and still more of the working woman, are the theme of so many newspaper articles and magazine stories. Even Walter Besant would admit that there is not much material for a grievance to be got in Messrs. Burroughs and Wellcome's factory.

